# Intraoperative robot-guided femoral osteotomy during robot-assisted total knee arthroplasty for knee osteoarthritis with extra-articular deformity: a case report

**DOI:** 10.3389/fsurg.2026.1910960

**Published:** 2026-08-28

**Authors:** Cai Liu, Yaqin Li, Hongyu Zhou, Hao Zhang, Xinwei Liu, Lianghu Zhao, Hao Tang, Dejie Zhou

**Affiliations:** 1 Department of Orthopedic Surgery, The Affiliated Hospital of Panzhihua University, Panzhihua, Sichuan, China; 2 Department of Physical Examination Center, Affiliated Hospital of Panzhihua University, Panzhihua, Sichuan, China; 3 Department of Orthopaedic Surgery, Beijing Jishuitan Hospital, Capital Medical University, National Orthopeadic Center of China, Beijing, China

**Keywords:** extra-articular deformity, femoral osteotomy, osteoarthritis, surgical robot, total knee arthroplasty

## Abstract

**Objective:**

To describe the short-term feasibility of using a CT-based robotic total knee arthroplasty (TKA) platform to plan and guide an intraoperative corrective femoral osteotomy when trial implantation revealed mechanical impingement in a patient with severe extra-articular femoral deformity.

**Design:**

A 60-year-old woman with end-stage knee osteoarthritis, 42° anterior femoral bowing, and a local 17° coronal valgus deformity underwent robot-assisted TKA. Corrective osteotomy was not included in the preoperative plan. After trial implantation produced a 20° extension deficit because of impingement between the polyethylene insert and the anterior femoral flange, the procedure was modified intraoperatively. The same TiRobot Recon-II platform (TINAVI, Beijing, China) was used to plan and guide a 20° anterior closing-wedge osteotomy at the deformity apex, followed by dual-plate fixation and definitive implantation of a posterior-stabilized resurfacing prosthesis.

**Results:**

The operation lasted 142 min, with an estimated blood loss of 200 mL. Full weight-bearing began on postoperative day 3. Knee range of motion was 0°−130° at 1 month and 0°−135° at 5 months. Postoperative full-length radiographs showed an HKA of 178.7°, corresponding to 1.3° of residual varus, and a MAD of 2.5 mm medial to the knee center. At 5 months, radiographs demonstrated osteotomy union, stable fixation, and maintained correction of limb alignment. The Knee Society Score improved from 29 to 91, and the Knee injury and Osteoarthritis Outcome Score pain subscale improved from 38 to 89. No wound, neurovascular, or implant-related complications were observed during the short follow-up period.

**Conclusions:**

In this single case, intraoperative conversion to robot-guided femoral osteotomy during robot-assisted TKA was technically feasible and was associated with favorable early clinical and radiographic outcomes. These findings support short-term feasibility only and do not establish superiority over planned simultaneous or staged strategies. Larger studies and longer follow-up are required.

## Introduction

Extra-articular deformities create substantial technical challenges during total knee arthroplasty (TKA), particularly when the deformity is severe, multiplanar, or close to the knee joint (1–4). The magnitude, direction, and location of the deformity can affect overall limb alignment, collateral-ligament balance, component position, and the amount of permissible intra-articular bone resection. Available strategies include intra-articular correction during TKA, planned same-stage corrective osteotomy with TKA, and staged deformity correction followed by delayed arthroplasty. A staged strategy permits confirmation of osteotomy union and may simplify the subsequent arthroplasty, but it requires separate procedures and prolongs the overall treatment course (5). Robot-assisted TKA has improved the ability to plan component position and assess gaps without relying on intramedullary guides (6–9). Additionally, robot-assisted TKA for extra-articular deformity without corrective osteotomy has been described and simultaneous TKA with corrective osteotomy has been reported in small case series and observational studies (2, 5, 6, 10).

The TiRobot Recon-II system (TINAVI, Beijing, China) provides CT-based three-dimensional planning, optical tracking, and intraoperative guidance for TKA bone resections. In the present case, femoral osteotomy was not prospectively planned; it was added as an intraoperative rescue modification after trial implantation revealed a mechanical extension block. To our knowledge, the potentially novel feature is the use of the robotic TKA platform itself to plan and guide the corrective femoral osteotomy during the same procedure, rather than the use of robot-assisted TKA or simultaneous osteotomy-TKA alone. This report describes the decision-making process, surgical workflow, and early clinical and radiographic outcomes.

## Case presentation

### Preoperative information

**Figure 1:**
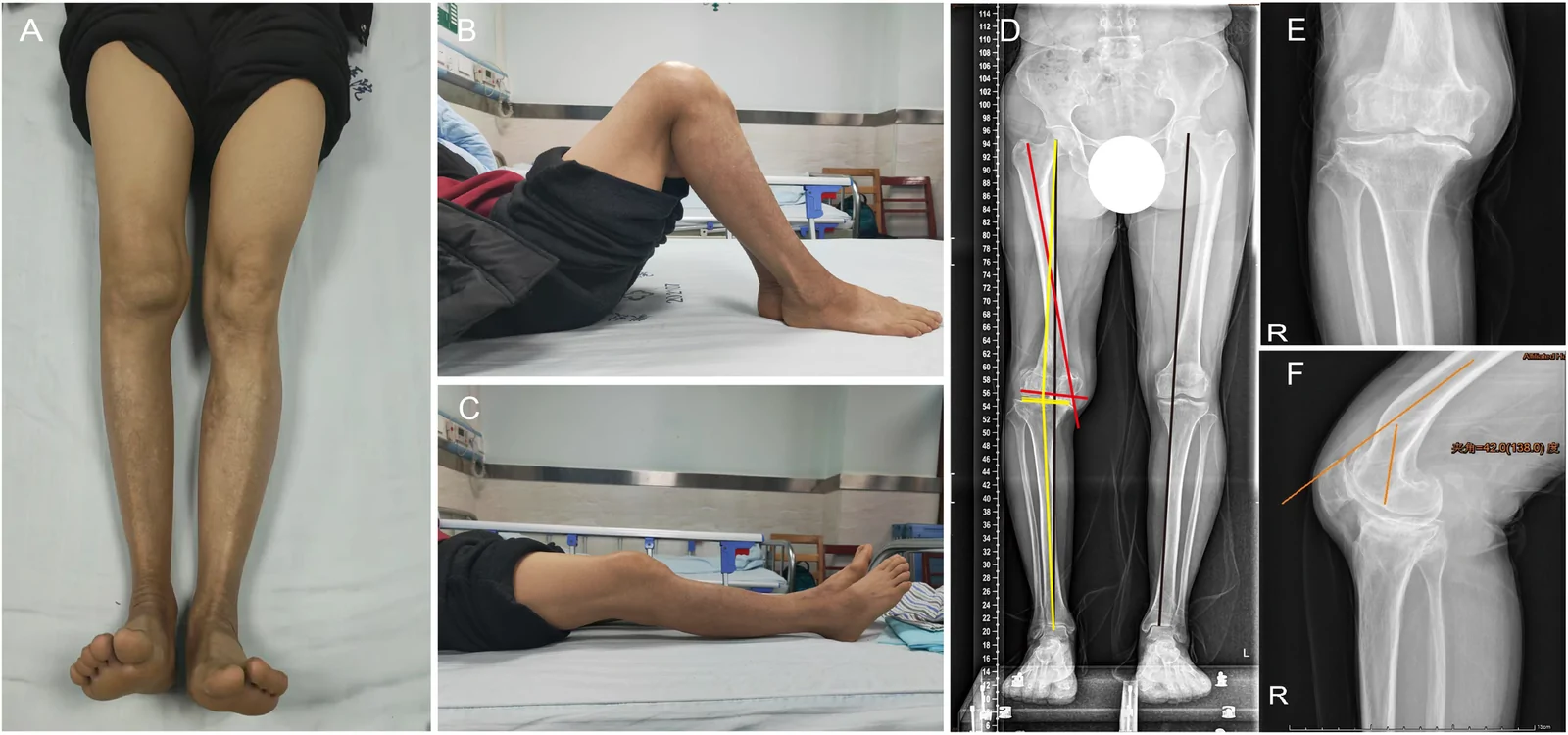
Preoperative clinical appearance and radiographic deformity analysis **(A)** clinical photograph showing overall varus alignment of both lower limbs. **(B)** Active knee flexion was limited to 110°. **(C)** Active extension showed an approximately 20° flexion contracture. **(D)** Standing full-length radiograph showing global varus alignment, with an HKA of 174.4° (5.6° varus) and a MAD of 20 mm medial to the knee center, despite local distal femoral valgus. The red and yellow arcs indicate the aLDFA (74.3°) and aMPTA (82.7°), respectively; the black line indicates the lower-limb mechanical axis. **(E)** Anteroposterior knee radiograph showing end-stage osteoarthritis and local distal femoral metaphyseal valgus. **(F)** Lateral radiograph showing severe anterior bowing of the distal femur; the deformity apex was localized approximately 67 mm proximal to the joint line on CT.

A 60-year-old female agricultural worker presented with a 40-year history of progressively worsening right knee pain, particularly during walking, squatting, rising from a seated position, and descending stairs. At 20 years of age, she sustained a traumatic injury that caused swelling and deformity of the distal right thigh and was treated conservatively with immobilization. Over subsequent decades, pain, deformity, and functional limitation gradually progressed. Physical examination demonstrated overall varus alignment of the right lower limb, a 20° flexion contracture, and painful active flexion limited to 110° (Figures 1A–C).

Standing anteroposterior radiographs demonstrated end-stage medial-compartment-predominant osteoarthritis, with joint-space obliteration, subchondral sclerosis, and large osteophytes. Formal deformity analysis distinguished local osseous deformity from global limb alignment. The limb was globally varus, with the mechanical axis passing medial to the knee center, despite a local 17° valgus angulation centered in the distal femoral metaphysis. The anatomical lateral distal femoral angle (aLDFA) was 74.3°, and the anatomical medial proximal tibial angle (aMPTA) was 82.7°. The preoperative hip-knee-ankle angle (HKA) was 174.4°, corresponding to 5.6° of global varus alignment, and the mechanical-axis deviation (MAD) was 20 mm medial to the knee center. These measurements confirmed global varus alignment despite the local distal femoral valgus deformity. Lateral radiographs showed approximately 42° of anterior angulation of the distal femur, and CT localized the deformity apex approximately 67 mm proximal to the joint line (Figures 1D–F). The initial plan was robot-assisted TKA with a primary resurfacing implant, without corrective osteotomy, because CT-based planning suggested that coronal component position and balanced gaps could be achieved without an intramedullary femoral guide. The limited preoperative extension deficit was considered potentially manageable by intra-articular resection and soft-tissue balancing. Corrective osteotomy was therefore not scheduled preoperatively. The clinical chronology is summarized in Table 1.

**Table 1:** Clinical timeline.

Time/phase	Clinical or intraoperative finding	Decision, intervention, or outcome
40 years before presentation	Traumatic distal femoral injury treated with immobilization; persistent deformity developed.	Progressive pain and functional limitation over subsequent decades.
Preoperative assessment	End-stage knee osteoarthritis; HKA 174.4° (5.6° varus); MAD 20 mm medial; local 17° distal femoral valgus; aLDFA 74.3°; aMPTA 82.7°; and 42° sagittal antecurvatum.	Robot-assisted primary TKA planned without corrective osteotomy because coronal alignment and gaps appeared manageable without an intramedullary guide.
Intraoperative TKA phase	Robot-guided TKA cuts and trial implantation produced balanced gaps but an unexpected 20° extension deficit caused by insert-anterior flange impingement.	TKA-alone plan judged mechanically unacceptable.
Intraoperative modification	The TKA planning module was repurposed to define a 20° anterior closing-wedge osteotomy at the deformity apex.	Robot-guided osteotomy, reduction, dual-plate fixation, repeat trialing, and definitive cemented TKA.
Postoperative days 2–3	Postoperative HKA 178.7° (1.3° residual varus), MAD 2.5 mm medial, stable implants, and satisfactory osteotomy apposition.	Brace-assisted ambulation on day 2; full weight-bearing on day 3.
1 month	Range of motion 0°−130°; stable fixation and early healing.	Continued progressive rehabilitation.
5 months	Range of motion 0°−135°; KSS 91; KOOS pain 89; radiographic union and maintenance of the corrected near-neutral alignment; no recorded complication.	Early clinical and radiographic follow-up completed.

### Overview of the surgical process

An image-based, real-time tracking TiRobot Recon-II system was used. A preoperative hip-knee-ankle CT scan was obtained to construct a three-dimensional model for TKA planning and robotic execution. Robot-assisted TKA was performed first according to the original plan. A posterior-stabilized total knee system (Legion, Smith & Nephew, Memphis, TN, USA; femoral size 3, tibial size 3) was provisionally selected. Planned component parameters were 22° of femoral flexion to avoid anterior notching, 0° femoral varus, 8° femoral external rotation, 3° tibial varus, and 5° posterior tibial slope. After trial implantation, the knee demonstrated an unexpected 20° extension deficit despite approximately 20-mm flexion and extension gaps. Direct inspection showed impingement between the polyethylene insert and the anterior femoral flange rather than a simple soft-tissue imbalance.

As additional distal femoral resection would have enlarged the extension gap and risked instability, while further sagittal reorientation of the femoral component would have increased the risk of anterior cortical notching, the surgical plan was modified intraoperatively. A 20° anterior closing-wedge osteotomy was designed at the deformity apex by repurposing the three-dimensional TKA planning module. The target was to eliminate the mechanical impingement and restore extension, rather than to correct the entire 42° sagittal deformity, which could have produced hyperextension in the setting of long-standing soft-tissue adaptation. The osteotomy was executed under robotic guidance, reduced, and stabilized with medial and lateral locking compression plates.

### Detailed surgical process

Spinal anesthesia was administered with the patient in the supine position. Cefazolin (2 g intravenously) and tranexamic acid (100 mL solution) were administered 30 min before incision. A proximal thigh tourniquet was applied and inflated to 240 mmHg. A standard anterior midline incision and medial parapatellar arthrotomy were used. Optical trackers were fixed to the medial distal femur and mid-tibia. CT-based registration was completed with intraoperative fluoroscopic verification, and the reported registration error was 0.3 mm.

**Figure 2:**
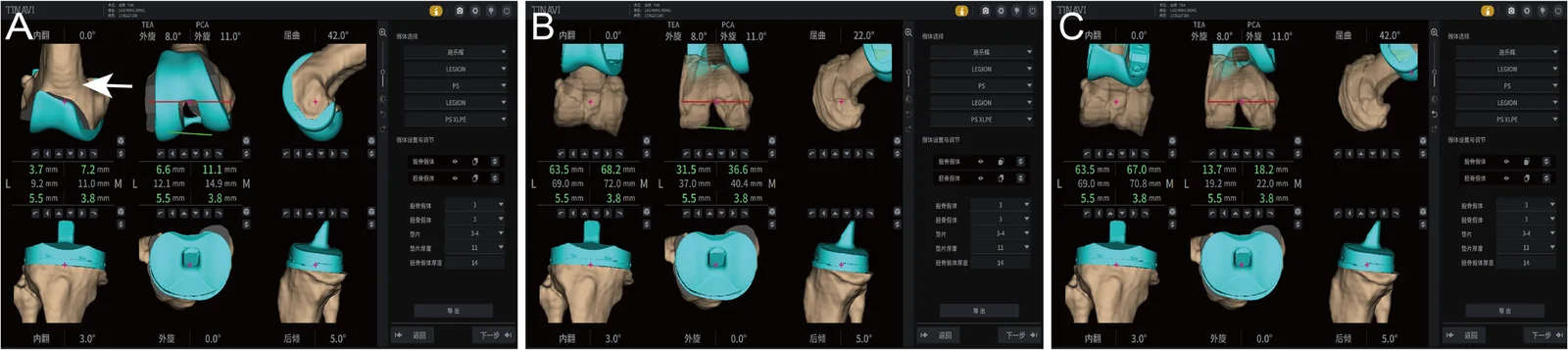
Robotic TKA planning and intraoperative osteotomy-plane design **(A)** alternative 20° sagittal reorientation of the virtual femoral component, demonstrating disappearance of the simulated notch. **(B)** Step 1 of osteotomy planning: the virtual femoral component was shifted proximally by 59.8 mm and extended by 20° to define the first cutting plane. **(C)** Step 2 of osteotomy planning: the virtual component was returned to its original sagittal orientation to define the second convergent cutting plane.

**Figure 3:**
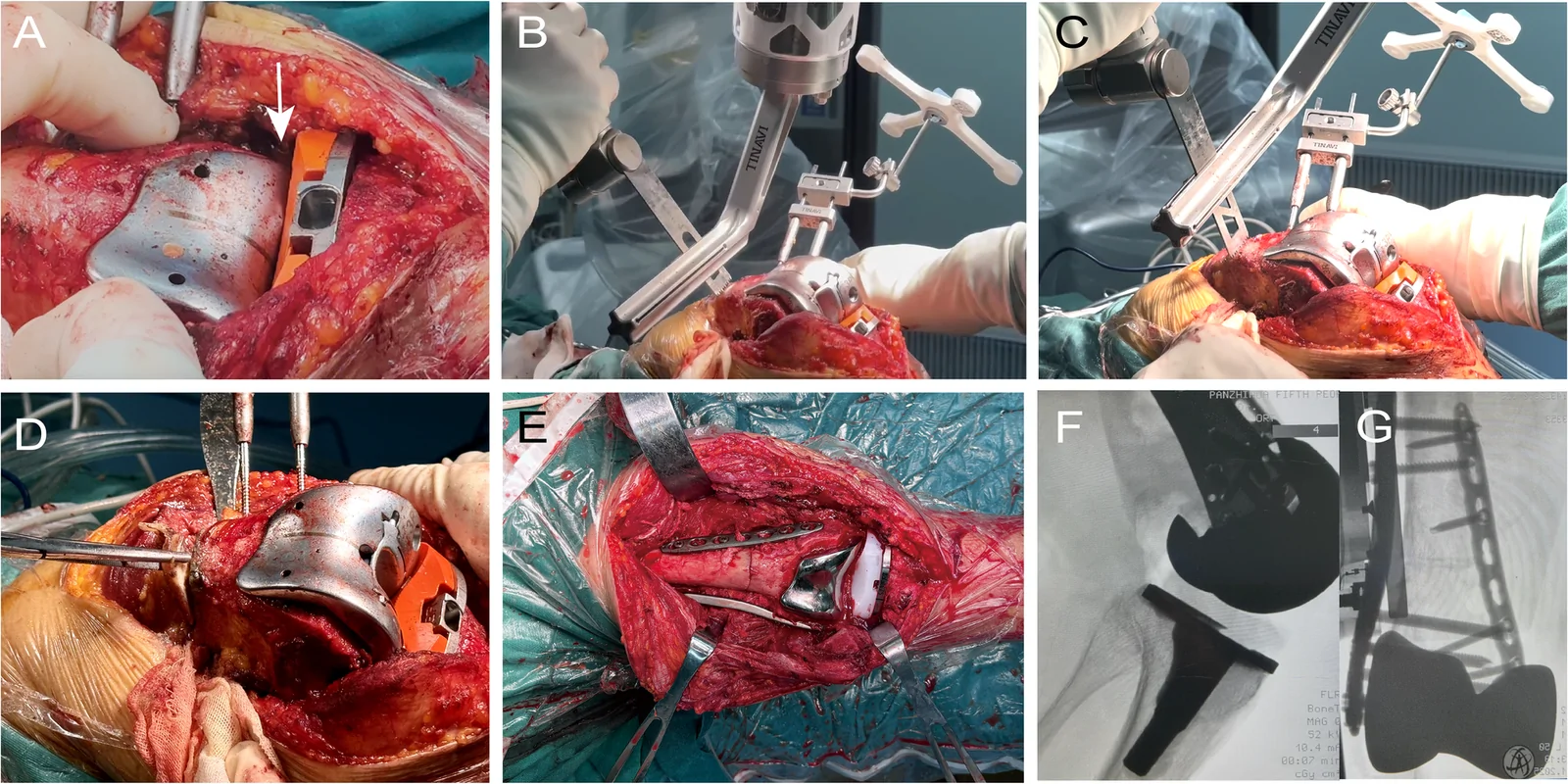
Intraoperative rescue osteotomy and fixation sequence **(A)** after TKA trial implantation, the knee remained in approximately 20° of fixed flexion because of impingement between the polyethylene insert and the anterior femoral flange. **(B)** First robot-guided osteotomy cut performed at the deformity apex according to the proximally shifted and 20°-extended virtual component plane. **(C)** Second osteotomy cut performed after the virtual component was returned to its original sagittal orientation. **(D)** Removal of the anterior wedge-shaped bone fragment. **(E)** Reduction of the osteotomy and fixation with lateral distal femoral and medial straight locking compression plates. **(F,G)** Final fluoroscopic images confirming osteotomy reduction, plate and screw position, and stable TKA component fixation.

Robot-guided bone resections for TKA were performed first. Femoral resection depths were 3.7 mm laterally and 7.2 mm medially, and tibial resection depths were 5.5 mm laterally and 3.8 mm medially. An 11-mm spacer produced balanced gap kinematics. Posterior femoral condylar resections measured 6.6 mm laterally and 11.1 mm medially, with a 5° posterior tibial slope (Figures 2B–D). Trial components were then inserted. Although the gaps appeared balanced, the knee remained in approximately 20° of fixed flexion because the insert impinged on the anterior femoral flange (Figure 3A).

A robot-guided anterior closing-wedge osteotomy was then performed at the deformity apex with a high-speed oscillating saw. During intraoperative planning, the virtual femoral component was shifted 59.8 mm proximally and extended by 20° in the sagittal plane to define the first osteotomy plane (Figure 3B). The virtual component was then returned by 20° to its original sagittal orientation to define the second plane (Figure 3C). The intervening wedge-shaped bone fragment was removed (Figure 3D). After reduction, satisfactory cortical apposition and correction were confirmed. Fixation was achieved with a lateral distal femoral locking compression plate and a medial straight locking compression plate (Figure 3E). Repeat trialing demonstrated full extension, stable kinematics, and satisfactory patellar tracking. The definitive components were cemented with gentamicin-loaded bone cement. Fluoroscopy confirmed reduction of the osteotomy, plate and screw position, and stable prosthetic fixation (Figures 3F,G).

An 80-mL multimodal periarticular analgesic mixture containing 200 mg ropivacaine, 10 mL flurbiprofen axetil, 50 mL normal saline, and five drops of epinephrine was infiltrated around the operative field. After pulse lavage, a drain was placed and the wound was closed in layers. Total operative time was 142 min and estimated blood loss was 200 mL. Five fluoroscopic images were acquired to confirm plate and screw position and the recorded radiation dose was 1.3 mSv.

### Postoperative course

Graduated compression stockings and intermittent pneumatic compression were used for venous thromboembolism prophylaxis. Subcutaneous enoxaparin was initiated 8 h after surgery. A postoperative dose of cefazolin (2 g intravenously) was administered, and tranexamic acid (100 mL solution) was infused to reduce blood loss. The drain was removed after 24 h. Diclofenac sodium enteric-coated tablets (0.1 g twice daily) were prescribed for analgesia.

**Figure 4:**
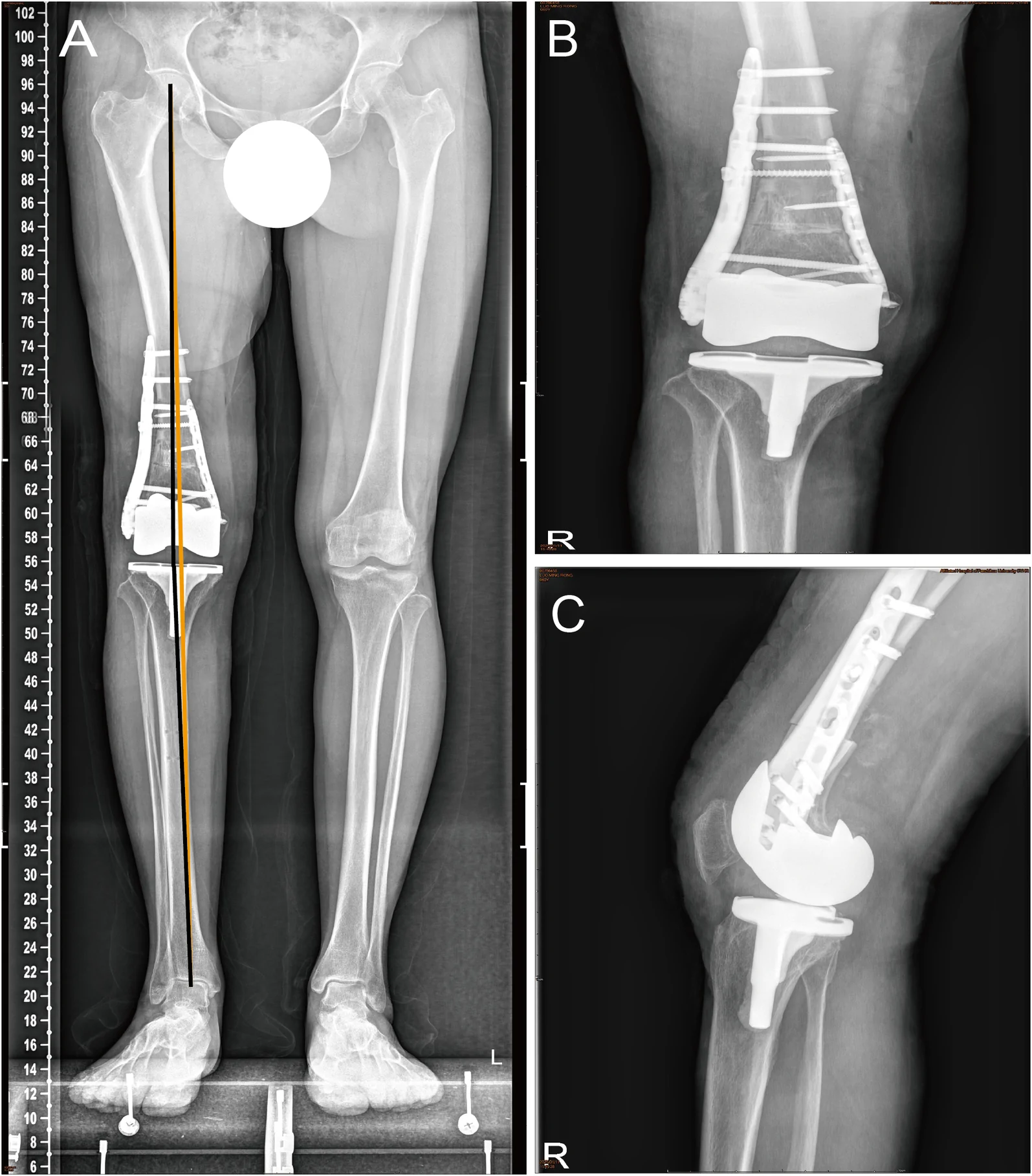
**Immediate postoperative radiographs (A)** full-length postoperative radiograph showing correction toward near-neutral alignment: HKA improved from 174.4° preoperatively to 178.7° postoperatively (1.3° residual varus), and MAD decreased from 20 mm to 2.5 mm medial to the knee center. The TKA components and fixation hardware are satisfactorily positioned. **(B,C)** Anteroposterior and lateral radiographs showing correction of the femoral antecurvatum and satisfactory apposition of the osteotomy surfaces.

On postoperative day 2, the patient began ambulation with a protective brace. Full-length radiographs demonstrated correction of the global varus alignment, with a postoperative HKA of 178.7° (1.3° residual varus) and a MAD of 2.5 mm medial to the knee center, compared with 174.4° and 20 mm medial preoperatively. The prosthesis and fixation hardware were satisfactorily positioned, the femoral antecurvatum was corrected, and the osteotomy surfaces showed satisfactory apposition (Figures 4A–C).

At the 1-month follow-up, full weight-bearing without an assistive device was allowed, and the active knee range of motion was 0°−130°. The patient could walk on level ground with only a mild residual limp. Radiographs showed stable component and plate fixation, no evidence of loosening, and early osteotomy healing with blurring of the osteotomy line.

**Figure 5:**
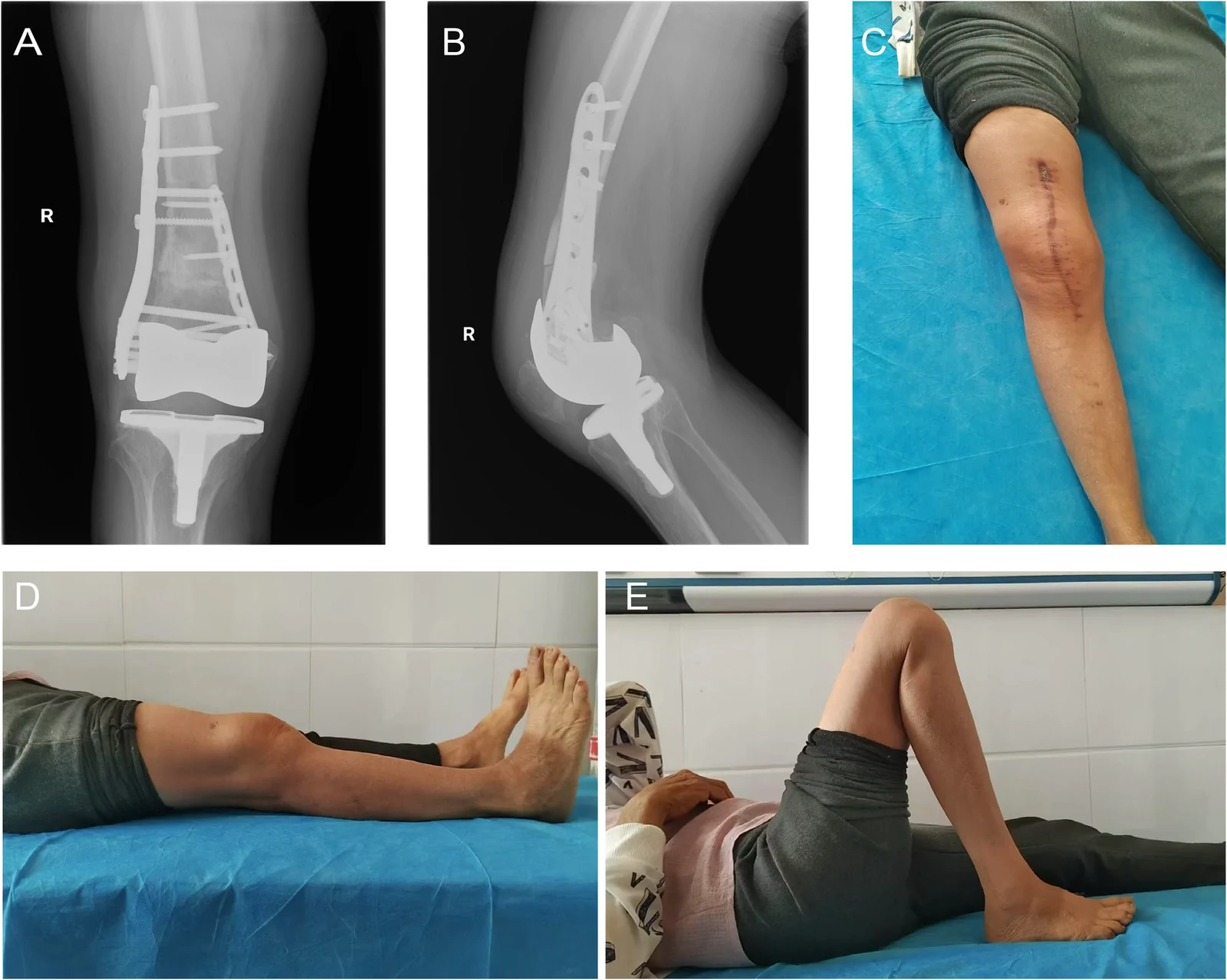
Radiographic and functional findings at 5 months **(A,B)** radiographs showing maintained prosthetic and plate fixation with progressive bridging and union of the osteotomy. **(C)** Healed incision without signs of infection. **(D,E)** Active extension and flexion of 0°−135°.

At the 5-month follow-up, gait and stair-climbing ability had improved, and active knee range of motion was 0°−135°. The Knee Society Score improved from 29 to 91, and the Knee injury and Osteoarthritis Outcome Score pain subscale improved from 38 to 89. No wound, neurovascular, or implant-related complication was observed. Radiographs demonstrated progressive bridging and union of the osteotomy with maintained correction of the postoperative near-neutral limb alignment (Figure 5).

## Discussion

This case should be interpreted as an intraoperative conversion or rescue strategy rather than a prospectively planned same-stage TKA-osteotomy procedure. The preoperative plan was robot-assisted TKA alone. Corrective femoral osteotomy was added only after trial implantation produced a mechanical extension block that could not be acceptably resolved by component repositioning or additional intra-articular resection. The principal finding is therefore the technical feasibility of using the robotic TKA platform to rapidly redesign and guide a corrective osteotomy during the same operation.

The novelty of the case is limited and specific. Robot-assisted TKA for extra-articular femoral or combined femoral-tibial deformity has previously been reported without corrective osteotomy (6, 7). Similarly, simultaneous TKA and corrective osteotomy have been described using conventional planning and instrumentation (2, 10, 11). To our knowledge, the distinctive feature of the present case is the intraoperative use of the robotic TKA planning environment to define and guide the corrective femoral osteotomy after an unanticipated trial-component impingement. This distinction avoids implying that either robot-assisted TKA or simultaneous osteotomy-TKA is itself unprecedented.

### Deformity analysis and intraoperative decision-making

Local deformity and overall limb alignment must be described separately. In this patient, the distal femur had a local 17° valgus angulation, whereas the standing limb was globally varus, with an HKA of 174.4° (5.6° varus) and a MAD of 20 mm medial to the knee center. Global alignment reflected the combined effects of femoral geometry, proximal tibial varus, and articular degeneration. The aLDFA of 74.3° and aMPTA of 82.7° characterized the local femoral and tibial geometry, whereas HKA and MAD quantified overall limb alignment (12, 13). Postoperatively, the HKA improved to 178.7° (1.3° residual varus) and the MAD decreased to 2.5 mm medial, indicating correction toward near-neutral mechanical alignment. In the coronal plane, the robotic plan suggested that acceptable component position and gap balance could be achieved intra-articularly (14–18). In the sagittal plane, however, the 42° antecurvatum created a geometric conflict that became apparent only after trialing. Because the preoperative extension deficit was limited to 20°, the team initially attempted TKA without osteotomy. After trialing increased the fixed-flexion position to 20° and direct inspection demonstrated implant-bone impingement, a 20° corrective osteotomy was selected to restore functional extension. Full correction of the 42° bony deformity was deliberately avoided because long-standing soft-tissue adaptation could have resulted in postoperative hyperextension. Alternative osteotomy techniques, including lateral condyle sliding osteotomy, have also been described for selected extra-articular femoral deformities, although they do not directly address severe sagittal deformity (19).

### Comparison with staged and planned same-stage strategies

Staged deformity correction permits isolated correction of alignment, confirmation of osteotomy union, and later TKA under more conventional anatomy. It may be preferable when substantial multiplanar correction, lengthening, soft-tissue reconstruction, or gradual correction is required. In a staged series of 27 patients with 30 deformities, Wallace et al. reported union in all cases and no transfusion, nerve palsy, or compartment syndrome, however, only 12 patients proceeded to primary or revision TKA during a mean 3.1-year follow-up, illustrating the longer and heterogeneous treatment pathway (20). Planned same-stage procedures avoid a second anesthetic and allow combined rehabilitation, but they also combine the risks of arthroplasty, osteotomy, fixation failure, nonunion, infection, stiffness, and thromboembolism. In 26 knees treated with same-stage TKA and tibial osteotomy, the mean International Knee Society score improved from 70 to 170 and flexion from 98° to 107° at a mean 9 years, only one patient developed major skin necrosis and infection requiring implant removal (2). In six same-stage femoral osteotomy cases, the mean score improved from 46 to 161 and flexion from 95° to 107°, with one deep-vein thrombosis and one case of stiffness requiring manipulation (11). A recent observational series of 10 simultaneous procedures reported improvement in the Knee Society Score from 32.3 to 79.4 at a mean follow-up of 49.2 months, with no major complications but two planning errors (10). Nevertheless, the available evidence remains limited due to the small number of studies and the lack of comparative research. The early recovery observed in the present patient should not be interpreted as evidence of superior functional recovery or a lower risk of complications compared with staged surgery.

### Role and limits of robotic guidance

The robotic platform was useful in three respects. First, it avoided intramedullary femoral referencing, which was impractical because of the distal femoral deformity. Second, CT-based planning and quantitative gap assessment supported component positioning with a primary resurfacing implant. Third, the virtual femoral component was repurposed intraoperatively to define two convergent osteotomy planes at the deformity apex. However, the reported 0.3-mm registration error reflects only the accuracy of image-to-patient registration and should not be interpreted as the accuracy of the executed osteotomy. The actual angular and translational errors of osteotomy execution were not quantified. Accordingly, claims of submillimeter osteotomy precision or superiority over manual osteotomy are not supported by this case. The system also lacked a dedicated extra-articular osteotomy module, making the workflow an off-label adaptation of the TKA planning function.

### Rationale for osteotomy and proposed selection criteria

Once impingement was identified, the alternatives included further distal femoral resection, sagittal repositioning of the femoral component, use of a more constrained implant, or corrective osteotomy. Additional distal resection would have enlarged the extension gap and risked ligament imbalance, excessive component extension would have increased anterior notching, and greater implant constraint would not have directly removed the extra-articular bony conflict. The osteotomy therefore provided a means to restore extension while preserving a primary posterior-stabilized resurfacing implant. Based on this experience, intraoperative conversion to osteotomy should be considered only when the deformity apex is accessible, bone stock permits stable fixation, the mechanical block is clearly defined, the required correction can be planned without jeopardizing neurovascular structures, and a surgical team experienced in both complex arthroplasty and deformity correction is available. Preoperative consent should include the possibility of osteotomy whenever severe extra-articular deformity may create an intraoperative conflict, even when TKA alone is initially planned.

### Limitations

This report has several limitations. First, it describes a single patient with only 5 months of follow-up, which is insufficient to evaluate the durability of deformity correction, late nonunion, plate-related symptoms, aseptic loosening, implant survivorship, or other delayed complications. Accordingly, the findings should be interpreted as evidence only of short-term feasibility and early clinical outcomes. Second, the accuracy of osteotomy execution was not quantitatively assessed by comparing the achieved correction with the intraoperative plan, and no control group was available for comparison. Third, the robotic platform lacked a dedicated osteotomy planning module, requiring the virtual femoral component to be repurposed to define the osteotomy planes. Forthermore, the present case involved a predominantly sagittal femoral deformity, more complex multiplanar deformity or tibial deformitis may require substantially different planning strategies, fixation techniques, and rehabilitation protocols. Therefore, longer-term follow-up and prospective studies are needed before this workflow can be considered beyond carefully selected cases.

Despite these limitations, the case lilustrates how intraoperative kinematic assessment can identify a mechanical abnormality that was not fully appreciated on static preoperative imaging and how a robotic planning platform can facilitate a corrective osteotomy during the same surgical procedure. This finding should be regarded as a preliminary technical proof of concept rather than evidence that robot-assisted rescue osteotomy is superior to either planned single-stage or staged treatment.

## Conclusion

In this patient, intraoperative robot-guided femoral osteotomy performed during robot-assisted TKA was technically feasible and was associated with osteotomy union, restoration toward near-neutral mechanical alignment (postoperative HKA 178.7° and MAD 2.5 mm medial), and favorable early functional recovery at 5 months. This procedure represented an unplanned intraoperative salvage strategy rather than a prospectively planned combined intervention. Consequently, no conclusions can be drawn regarding its long-term safety, implant survival, or superiority over either staged or planned same-stage procedures can be made from this report.

## Authorship declaration

All listed authors meet the authorship criteria of the International Committee of Medical Journal Editors and approved the final manuscript.

## Data Availability

The raw data supporting the conclusions of this article will be made available by the authors, without undue reservation.
